# DDSM: Design-Oriented Dual-Scale Shape-Material Model for Lattice Material Components

**DOI:** 10.3390/ma15217428

**Published:** 2022-10-23

**Authors:** Chao Feng, Rui Yang, Bin Niu, Xiangpeng Meng

**Affiliations:** School of Mechanical Engineering, Dalian University of Technology, Dalian 116024, China

**Keywords:** lattice material components, dual-scale model, non-manifold topology, implicit representation

## Abstract

This paper proposes a new CAD model for the design of lattice material components. The CAD model better captures the user’s design intent and provides a dual-scale framework to represent the geometry and material distribution. Conventional CAD model formats based on B-Rep generate millions of data files, which also makes design intent and material information missing. In the present work, a new shape-material model for lattice material components is proposed. At the macroscopic scale, a compact face-based non-manifold topological data structure is proposed to express the lattice shape-material information without ambiguity. At the microscopic scale, implicit function is adopted for the representation of lattice material components. Numerical experiments verify that the proposed CAD model provides a powerful support for design intent with minor space costs. Meanwhile, the representation method supports solid modeling queries of geometric and material information on each scale.

## 1. Introduction

Different from conventional components made of the same material uniform, lattice material component (LMC) refers to objects with spatially homogeneous material and different periodic microstructures. The LMC is widely used in the lightweight design of aerospace structures due to its excellent mechanical properties (e.g., high specific strength, stiffness [1] and shock absorption [2]). Based on homogenization theory, the LMC is regarded as a component consisting of multiple homogeneous materials at the macroscopic scale. At the microscopic scale, the LMC is composed of a periodic array of different types of unit cells. The LMC provides more design freedom for designers to further control the distribution of equivalent properties of materials at the macroscopic scale by controlling both material compositions and their microstructures [3,4].

The CAD model, as an explicit reflection of the user’s design intent, is the basis for subsequent simulation and manufacturing. It is crucial to propose a new CAD model for the LMC dual-scale characteristics. Conventional CAD models are not efficient at representing lattice structures [5]. Since the manufacturing of lattice structures is mostly achieved by additive manufacturing [6], about 80% of lattice files are stored in stereolithography format (STL) [7]. However, the triangular mesh-based CAD model can only reflect the geometric information of the components discretely, ignoring the dual-material information (i.e., homogenized equivalent materials and manufacturing materials). In addition, the STL file will be useless when the microstructures need to be modified. Consequently, a CAD model that can completely and efficiently represent and handle the shape-material information of the LMC is extremely critical. The shape information includes geometric information and topological relationships. Additionally, the material information includes homogenized equivalent materials and manufacturing materials. Therefore, there are two basic challenges which require a solution to the CAD model. Firstly, the model needs to reflect clear design intent. Secondly, it needs to contain the complete shape-material information with minor space costs.

Spatial decomposition as a CAD model scheme includes the voxel model and the mesh model [8]. A voxel-based method was proposed for the generation of trimmed lattice structures [9]. The overall material distribution is determined by specifying a material composition to each voxel directly. However, the problems with this method are the large memory overhead [10] and the complexity of the material information query [11]. In terms of the volume mesh-based model, the shape-material model is represented as a collection of polyhedrons [12,13,14]. Each polyhedron is represented as a list of vertices, where the geometric position, as well as the material composition information, can be stored. However, the homogenized equivalent material information makes internal boundary vertices appear ambiguous in the storage process.

In contrast to the spatial decomposition, the analytical models separate geometric and material information. The representations based on the analytical models can be divided into two categories. In the first analytical model representation, the boundary representation (B-Rep) is used to represent geometric information. The material information is described by some specific functions (e.g., explicit functions [15] and distance function [16]). For example, The B-spline volume is utilized to represent the shape-material model. Additionally, the material information of the model is attached to each control point [17,18]. Second, the implicit functions are used to represent point set geometry and the material distribution. Specifically, the implicit function-based models reduce memory consumption compared to the STL files [19]. However, the dual-material information distributed on two scales is not represented by such an analytical model.

The composite model is used to represent components with two or more types of material distributions. The composite model is proposed based on the idea of spatial decomposition. There are two main modeling methods as follows. Firstly, the component is modeled by regularized operators of sub-objects, such as difference, intersection, union, etc. However, this approach faces the problem of heavily data redundant [20], cumbersome for regularized Boolean operations [21], and material ambiguity [22]. Secondly, the non-manifold geometric representation is used to produce a composite model [23]. However, it requires complex data structures and algorithms to construct the topological relationships for sharing the same boundary. 

According to the existing material component modeling methods, the LMC CAD model representation has the following problems to be solved.

1. Lack of dual-material model representation: Due to the dual-material properties on the lattice CAD model, the geometric model based on STL file format does not provide a complete representation of the material information. Moreover, this renders the model computationally expensive for the material query. Therefore, there is an urgent need to propose a dual-scale shape-material model, which supports material information queries on two scales.
2. Large storage cost. As mentioned before, most lattice structures are stored in the STL format. A large number of hollowed-out structures produce huge triangular facets to store geometry information. These triangular facets take up large memory and make the information redundant extremely.
3. Missing design intent: In the design-oriented process, designers prefer to focus on the overall equivalent material properties (elastic modulus, Poisson’s ratio, etc.) embodied in design domain, rather than on the specific configuration and manufacturing material of the unit cell. However, the existing CAD tools and model representation format (e.g., Delaunay triangulation) lack equivalent macroscopic material properties, which makes CAD models poorly designable and revisable.

In this paper, we strive to develop a new CAD shape-material model to solve the above problems. We refer to our model as Design-oriented Dual-scale Shape-material Model (DDSM). The main contributions of this work are summarized as: First, a new model representation method is proposed to meet the modern LMC design needs. Second, a new non-manifold topological data structure is used to represent macroscopic shape-material model. Third, an implicit function-based representation of truss-like struts is introduced at the microscopic scale. Consequently, this new dual-scale representation provides more intuitive the design intent and more efficient storage than conventional CAD model.

The remainder of the paper is organized as follows. Section 2 presents a mathematical framework of DDSM. Section 3 proposes a new data structure for finite non-manifold characteristic at the macroscopic scale. Section 4 provides geometric representation based on implicit functions at the microscopic scale. Section 5 proposes the DDSM formulation and advantages. Section 6 is the conclusions of this research.

## 2. Mathematical Frameworks for the DDSM

**Figure 1:**
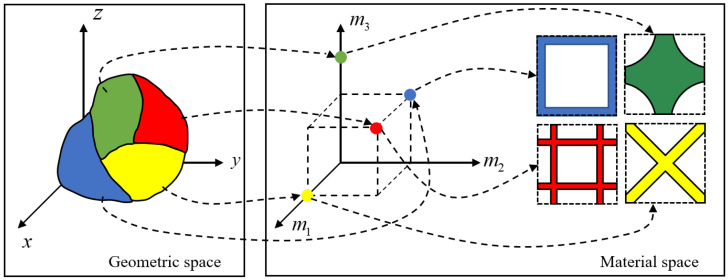
The CAD model of the ideal lattice material component.

The CAD models of lattice structure are usually converted into STL files. Therefore, it is less convenient for designers to capture the design intents. Firstly, for the design process of the LMC, the ideal design should be optimized for performance based on the specific functional requirements. Then, each sub-design domain obtains the optimal material distribution such as elastic modulus [24,25], Poisson’s ratio [26], shear modulus [27] etc. Finally, the unit cell configuration is chosen based on macroscopic material distribution. The ideal design process for the LMC is shown in Figure 1. The corresponding CAD model needs to reflect the ideal design process of the LMC and not just the discrete geometric information.

Depending on the design intent of the lattice material component, a material space is introduced based on the geometric space. The material space is made up of equivalent material properties with corresponding unit cell configurations. The LMC model representation is essentially a product space consisting of the geometric space as the base space and the material space as the bundle space. Thus, the information of any point in space can be expressed by a dual pair of geometric space and material space. The dual pairs realize the partitioning of the geometric space. The partitioning results in a collection of sub-objects, and each sub-object corresponds to a material space. From the perspective of topology, the point set partitioning forms a finite number of continuous open subsets, and the intersection of each open subset is empty. The union of all open subsets is the original geometric region. Therefore, the CAD model S for the LMC can be composed of several sub-objects. Each sub-object is described by geometric information and material information, i.e.,

(1) $$S=\left\{S_{1},S_{2},\ldots ,S_{n}\right\}\;S_{i}=\left\{\left(P,M\right)|P\in \Omega_{g}^{\left(i\right)},M\in \Omega_{m}^{\left(i\right)},1\leq i\leq n\right\}$$

where $S_{i}$ is the *i*-th sub-object, P is the geometric description of the sub geometric space $\Omega_{g}^{\left(i\right)}$, and M is the material description in the sub material space $\Omega_{m}^{\left(i\right)}$.

In summary, the LMC CAD model describes geometric and material information in two dimensions: macroscopic scale and microscopic scale. The dual-scale CAD model needs to consider the material information while representing the geometric information on each scale. The correspondence in mechanical behavior between the two scales is expressed in terms of equivalent material properties computed by the unit cell [28,29].

## 3. Macroscopic Scale: A Compact Face-Based Topological Data Structure

### 3.1. Finite Non-Manifold Characteristic

**Figure 2:**
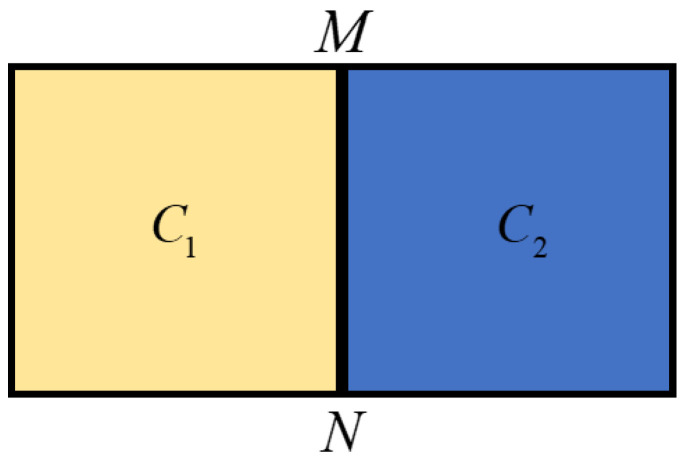
The ambiguity of inner boundary. MN is the edge shared by adjacent material spaces.

The geometric information, as the base space of the material information, is the key to the LMC CAD model. However, the conventional model representation based on Regular Set and Manifold Theory mathematically faces ambiguous representation of boundary materials as shown in Figure 2. For example, $C_{1}$ and $C_{2}$ correspond to different material space. Points on the internal boundary MN cannot define their equivalent material attribution when storing material information by B-Rep. The ambiguity of material information causes the typical non-manifold of CAD models at the macroscopic scale.

**Figure 3:**
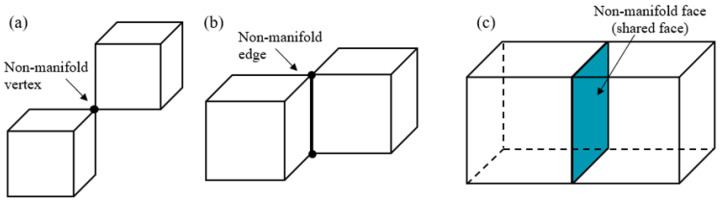
A non-manifold object formed by sharing (**a**) a vertex; (**b**) an edge; (**c**) a face.

According to Ellul [30] “in 3D, for the manifold to be valid, the neighborhood of each point within the sub-space must be able to be deformed into a sphere. Thus, self-intersecting surfaces are non-manifold”. It usually consists of a finite number of manifolds sharing boundaries as shown in Figure 3.

Due to the internal boundary material ambiguity, the LMC model appears as a “finite non-manifold”, i.e., it is simply limited to the presence of shared boundaries between material regions as shown in Figure 3c. Our goal is to design efficient and simple topological data structures for the finite non-manifold characteristic. The current non-manifold data structures are classified as the edge-based data structure and the face-based data structure. The edge-based data structures often utilize pseudo-manifold representation for non-manifold geometry. For example, a non-manifold shape with shared edges can be transformed into a manifold with two edges infinitely close to each other [31]. The face-based data structure was proposed for non-manifold and non-regular simplicial complexes. However, the storage of topological information in intermediate dimensions causes unnecessary memory overhead [32].

### 3.2. A compact Half-Face Data Structure Design

A well-defined CAD data structure defines the topological relationships of geometric entities completely. For the macroscopic LMC model, it is desirable to meet the following requirements:

- Generality: support for macroscopic non-manifold geometry CAD representations.
- Time efficiency: support specific topology queries without performing global search.
- Memory overhead: require a minimal amount of storage.

In this paper, we propose a mesh-based data structure to represent the LMC macroscopic model. The data structure establishes complete topological relationships. We refer to our data structure as the Compact Array-based Half-Face (CAHF) data structure oriented to finite non-manifold. First, we define several entity concepts. If a face has two incident elements, we refer to such face as twin half-face. A face without any twin is a border half-face. Vertices incident on only one element are called manifold border vertices.

**Figure 4:**
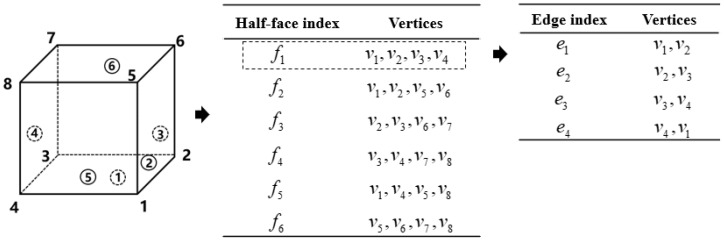
Local numbering conventions and implicit topology information for 3-D elements. The circled numbers correspond to local face IDs, and the unmarked ones correspond to local vertex IDs in the standard 3-D element.

The local numbering conventions in the CAHF data structure is shown in Figure 4. Moreover, the elements in the volume mesh carry more topology information. For example, the order of storing vertices implicitly expresses the order of faces(2-D) and edges(1-D), which does not be stored but referenced implicitly. This representation makes full use of the implicit topology information between vertices and faces within the element. Additionally, this effectively reduces memory overhead.

More specifically, each face ID is composed of a pair of numbers $\langle E_{id},F_{id}\rangle$, where $E_{id}$ denotes the element ID (starting from 1), and $F_{id}$ denotes the local face ID. The number of elements in a complex mesh model can be in the millions, and the element ID requires a large range of integer data. Additionally, the maximum local face ID is six. To avoid the space consumption caused by memory alignment, the face ID is coded as a single 32-bit unsigned integer. The element ID is stored in the first twenty-nine bits and the last three bits store the local face ID [33]. Therefore, about 500 million elements can be stored based on the half-face coding.

**Figure 5:**
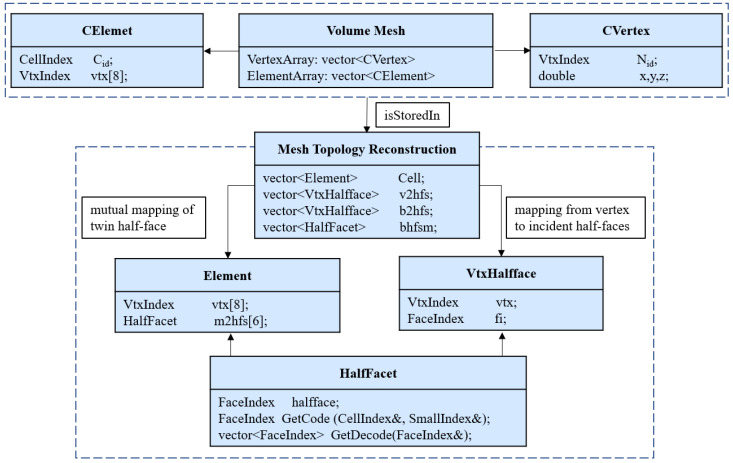
Class diagram of the CAHF volume mesh model. Let *CellIndex*, *FaceIndex* and *VtxIndex* denote the ID of element, half-face and vertex, respectively. *SmallIndex* is the local ID of the face. The type of these variables is unsigned int.

Based on the above topological conventions, the CAHF data structure oriented to finite non-manifold characteristic is proposed as shown in Figure 5. Volume meshes contain element connectivity and node coordinates. The element connectivity and node coordinates are used as input. These are stored in Class *Element*. Class *Element* also opens a one-dimensional array that stores the twin half-face of each face in the element. Class *HalfFacet* is an encoding and decoding operation for half-face information. Class *VtxHalfface* is used to store the topological relationships by Mesh Topology Reconstruction.

### 3.3. Macroscopic CAD Model Topology Reconstruction

For the DDSM macroscopic model to handle geometric information, it is desirable to reconstruct the topological relationships. Therefore, we define the arrays for the CAHF data structure similar to that in Dyedov et al. [34]:

- *m2hfs*: Map each half-face to the ID of its twin half-face;
- *v2hfs*: Map each non-manifold vertex to its incident half-face;
- *b2hfs*: Map manifold border vertex to its incident element ID and its local ID;
- *bhfsm*: Map each border half-face to its incident element ID and its local ID.

Specifically, the topology reconstruction consists of the following two algorithms. Algorithm 1 describes the identification of twin half-faces. The identification starts by traversing the faces on each element and storing the index of the largest vertex on each face in the temporary array *v2fs*. Then, the adjacent vertices of the largest vertex on each face are stored in the temporary array *v2adj*. Finally, the twin half-face is matched through two temporary arrays.
**Algorithm 1:** Construction of twin half-faces
**Input:** element connectivity, *CElement*
**Output:** mutual mapping of twin half-faces, *m2hfs*
**begin**
 Allocate the initial *Element* and define *m2hfs* zeros ** for each**
*e* in *CElement*
**do**
  *f*←traverse each face in *e*;   $ID_{max}$←get the maximum node identification on *f*;   set mapping $ID_{max}$ to the corresponding face into *v2fs*;   set mapping $ID_{max}$ to adjacent vertices into *v2adj*;  **end for**
 **for each**
*e* in *CElement*
**do**
  *f*←traverse each face in *e*;   **if**
*m2hfs (f) = 0*
**then**
   $ID_{max}$←get the maximum node identification on *f*;    get candidate faces of mutual mapping on *f*;    match twin half-face of *f* in *v2adj*;   **end if**
 **end for**
**end**

Algorithm 2 implements the mapping between the different dimensional entities. Specifically, non-manifold vertices are mapped in the array *v2hfs*. This is a one-to-many mapping. The manifold border vertices are stored in the array *b2hfs*. Meanwhile, it is useful to extract the geometric boundaries for numerical analysis. Therefore, the map *b2hfsm* is used to store all the manifold faces.
**Algorithm 2:** Construction of topological relationship
**Input:** element connectivity, *CElement*
  mutual mapping of twin half-faces, *m2hfs*
**Output:** vertex to incident half-faces, *v2hfs*
  border vertex to incident half-faces, *b2hfs*
  border half-faces mapping, *bhfsm*
**begin**
 Allocate array *marked* initialized to false and it is the same size as *m2hfs*;  **for each**
*e* in *CElement*
**do**
  *f* ←traverse each face in *e*;   **if not**
*m2hfs (f)*
**and not**
*marked(f)*
**then**
   set mapping vertices on face to *f* into *v2hfs*;   **end if**
 **end for**
 **for each**
*e* in *CElement*
**do**
  *f* ←traverse each face in *e*;   **if not**
*m2hfs(f)*
**then**
   set mapping *f* into *bhfsm*;    *v* ←traverse each vertex in *f*;    *V* ←set of elements incident on *v* in *v2hfs*;    **if not**
*V*
**then**
    append *f* to *b2hfs*;    **end if**
  **end if**
 **end for**
**end**

### 3.4. Topology Information Query

The purpose of reconstructing topological relationships is to enable efficient querying and processing of mesh information. We summarize the basic queries as follows.

**Figure 6:**
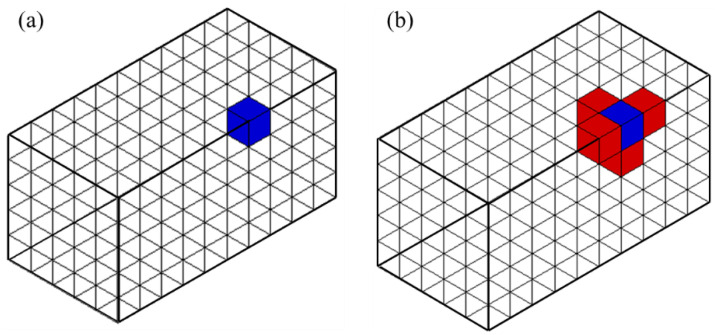
An example of a 3-D element adjacency query. For (**a**) a given blue element, it shows (**b**) adjacent red elements.

1. Adjacency query:

- Given an edge, return vertex-connected adjacent edges;
- Given a face, return edge-connected adjacent faces;
- Given an element, return face-connected adjacent elements;

Algorithm 3 describes adjacency query for the given element as an example. Figure 6 shows the example of the operation. The complete topological relationship supports the adjacent query time complexity of O(1).
**Algorithm 3:** Adjacency query for shared sub-entity
**Input:** element identification number, *elem*
  mutual mapping of twin half-faces, *m2hfs*
**Output:** adjacent three-dimensional entities, *cadj*
**begin**
 **for each** face *f* in *elem*
**do**
  **if**
*m2hfs (f)*
**then**
   *e*←decode to obtain face-connected adjacent elements;    append *e* to *cadj*;   **end if**
 **end for**
**end**

**Figure 7:**
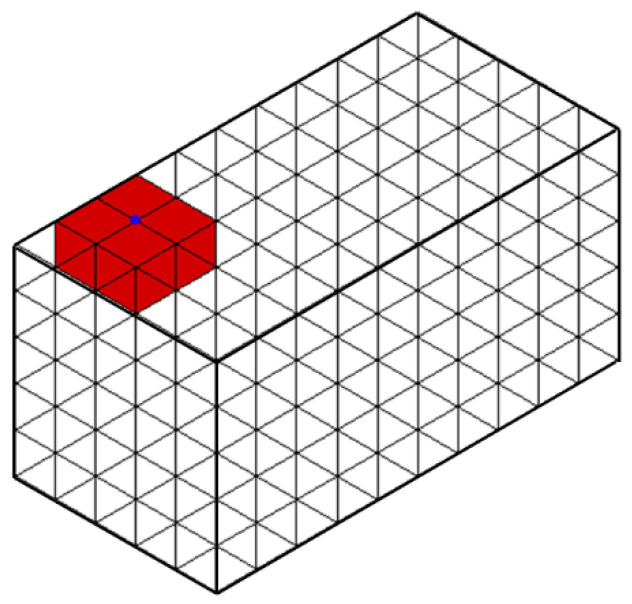
An example of incidence queries (red) for a non-manifold vertex (blue).

**Figure 8:**
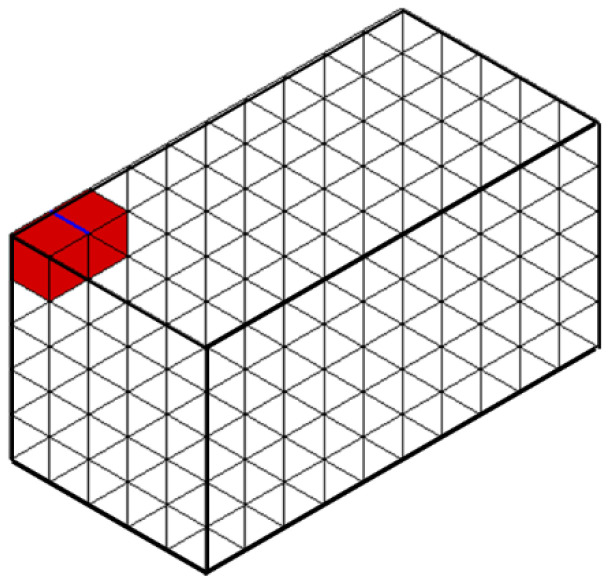
The example of incidence queries (red) for a given non-manifold edge (blue).

2. Incidence query:

- Given a vertex, return incident elements;
- Given an edge, return incident elements;

As an example, Algorithm 4 for querying the incident elements of an edge is as follows. Figure 7 and Figure 8 show examples of these operations.
**Algorithm 4:** Upscaling incidence query
**Input:** An edge defined by two vertices, *s*
  vertex to incident half-faces, *v2hfs*
  border vertex to incident half-faces, *b2hfs*
**Output:** incident three-dimensional entities, *cinc*
**begin**
 *v1*←vertex with larger ID in *s*;  *V1*←set of elements incident on *v1* found in *v2hfs(v)*;  **if not**
*V1*
**then**
  *V1*←set of elements incident on v1 found in *b2hfs(v)*;  **end if**
* v2*←adjacent vertex to *v1* in *s*;  *V2*←set of elements incident on *v2* found in *v2hfs(v)*;  **if not**
*V2*
**then**
  *V2*←set of elements incident on *v2* found in *b2hfs(v)*;  **end if**
 *cinc*←the intersection of *V1* and *V2*; **end**

The topology query results show that the proposed CAHF data structure is effective. It can achieve topology reconstruction and topology queries within a limited complexity. The CAHF exploits the implied topological relationships between geometries and greatly reduces the memory overhead. This data structure is a solution to the finite non-manifold characteristic of adjacent geometries sharing faces. Therefore, the LMC can be viewed as a component consisting of the volume mesh at the macroscopic scale. Each mesh corresponds to an equivalent material. The CAHF data structure constructs the complete topology relationships so that the boundary geometric information between different materials can be achieved easily.

## 4. Microscopic Scale: Lattice Unit Cell Based on Implicit Representation

### 4.1. Implicit Function of Truss-like Struts

At the microscopic scale, we propose an implicit representation of truss-like strut. In this work, the composition of the material at the microscopic scale can be considered as homogeneous. The CAD model based on implicit function greatly supports material evaluation. Specially, the shape is determined by the implicit function Φ(X). Given a geometric point $X_{i}$,$\Phi (X_{i})\leq 0$ means that material is present at that point. When $\Phi (X_{i})>0$, it means that there is no material at that point.

**Figure 9:**
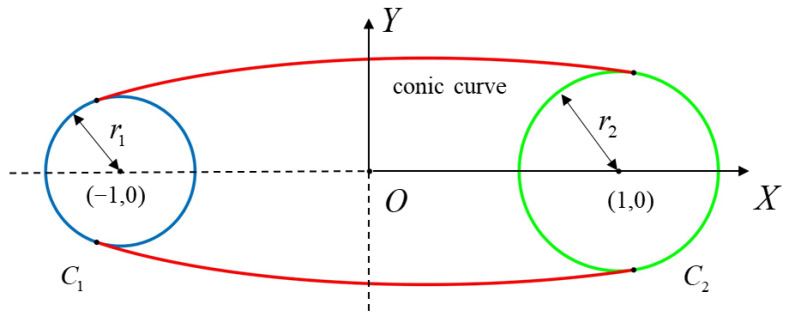
Construction of variable truss-like strut.

As shown in Figure 9, the shape of the strut can be regarded as a composite of two tangent circles *C*_1_, *C*_2_ and a conic curve. Given two points as the center of the circle and radius, the shape is entirely determined by the conic curve. In the modeling coordinate system (Figure 9), the shape can be represented by a quadratic equation after normalization (i.e., b=1):

(2) $$ax^{2}+by^{2}+2cx+d=0$$

and it becomes in the matrix form:

(3) $$\left[\begin{array}{ccc} x & y & 1 \end{array}\right]\left[\begin{array}{ccc} a & 0 & c\\ 0 & b & 0\\ c & 0 & d \end{array}\right]\left[\begin{array}{c} x\\ y\\ 1 \end{array}\right]=0$$

The circles *C*_1_, *C*_2_ are denoted as:

(4) $$C_{1}:{\left(x+1\right)}^{2}+y^{2}={\left(r-k\right)}^{2}$$

(5) $$C_{2}:{\left(x-1\right)}^{2}+y^{2}={\left(r+k\right)}^{2}$$

The symbols are defined as, $r=\left(r_{1}+r_{2}\right)/2$ and $k=\left(r_{2}-r_{1}\right)/2$. As the circles are tangent to the curve, we can calculate two parameters of the curve equation. Thus, we take *C*_1_ as an example from (2) and (4). Depending on Equation (6) obtained by the union, its discriminant will be zero. Therefore, we achieve:

(6) $$ax^{2}+2cx+d={\left(x+1\right)}^{2}-{\left(r-k\right)}^{2}$$

(7) $$d=\frac{1}{a-1}\left[{\left(c-1\right)}^{2}-\left(a-1\right){\left(r-k\right)}^{2}+\left(a-1\right)\right]$$

similar reasoning with circle *C*_2_ gives:

(8) $$d=\frac{1}{a-1}\left[{\left(c+1\right)}^{2}-\left(a-1\right){\left(r+k\right)}^{2}+\left(a-1\right)\right]$$

We can solve *c* from (7) and (8), and achieve the value of *d* into either (7) or (8):

(9) c=(a−1)kr

(10) $$d=\left(\frac{a}{a-1}\right)+\left[\left(a-1\right)r^{2}-1\right]k^{2}-r^{2}$$

**Figure 10:**
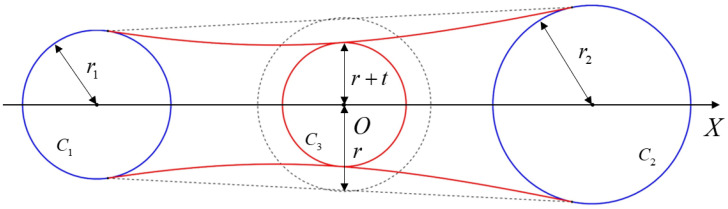
Profile curve controlled by offset parameter *t*.

The unknown constant *a* as a free input variable determines the unique strut type as shown in Table 1 [35]. However, it is not very intuitive for unit cell geometric design. Based on this, a new explicit geometric representation is proposed as shown in Figure 10. Specifically, we introduce a third circle *C*_3_ with center coordinates (0, 0) and an initial radius of *r*. Additionally, the offset parameter *t* is introduced as a substitute for *a*. The circle *C*_3_ with radius r+t is tangent to the curve. A positive offset parameter makes the strut elliptical, whereas a negative offset parameter makes the strut hyperbolic.

By introducing the offset parameter, the conic curve is precisely defined. Therefore, we replace *a* with the offset parameter *t*:

(11) $$a\left(t\right)=\frac{k^{2}-t^{2}-2rt}{k^{2}-t^{2}-2rt-1}$$

**Figure 11:**
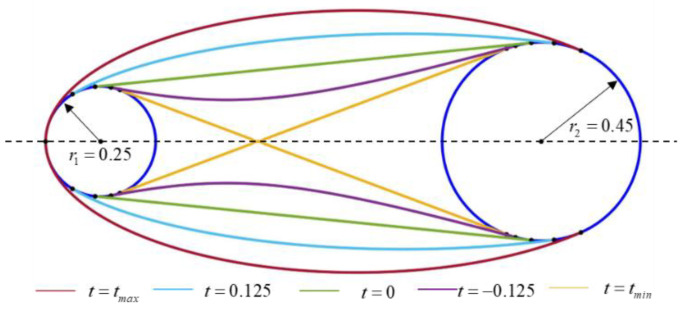
The strut shapes controlled by the offset parameter *t*.

As shown in Figure 11, the strut shapes controlled by the offset parameter *t* are discussed based on the given two circles. The maximum and minimum values of the offset parameter can be calculated as follows. When the tangency points are on the *x*-axis, the value of *t* will be the maximum. The maximum value of *t* can be calculated from (11).

(12) $$t_{m}{}_{ax}=-r+\sqrt{k^{2}+\left(r-k\right)\left(r+1\right)}$$

The value of *t* will be the minimum when the conic curve becomes an inner common tangent about circles *C*_1_, *C_2_*. The existence condition of the circle *C*_3_ also should be considered (i.e., $r+t_{m}{}_{in}>0$). We achieve:

(13) $$t_{m}{}_{in}=k-r$$

As analyzed above, we propose an implicit representation for the variable shape. The parameters *r_1_, r_2_* and *t* are used to replace *a*, *c*, *d* in the equation. Moreover, it is more intuitive and flexible to control the shape of the strut.

### 4.2. Generation of Lattice Unit Cell

The implicit function precisely represents the shape profile of the strut. A unit cell can be viewed as consisting of several struts. Thus, the microscopic CAD shape-material model is represented by the following steps. Firstly, the profile curve is rotated around the *x*-axis (Figure 9) to generate a 3D solid in the local modeling coordinate system (LCS) from (14).

(14) $$\left[\begin{array}{cccc} x & y & z & 1 \end{array}\right]\left[\begin{array}{cccc} a & 0 & 0 & c\\ 0 & 1 & 0 & 0\\ 0 & 0 & 1 & 0\\ c & 0 & 0 & d \end{array}\right]\left[\begin{array}{c} x\\ y\\ z\\ 1 \end{array}\right]=0$$

which can be simplified as:

(15) $$XPX^{T}=0$$

**Figure 12:**
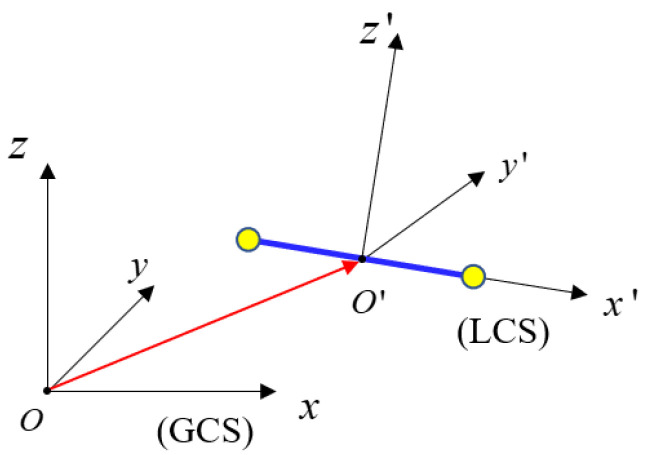
The illustration of strut expressed in LCS and GCS.

Next, the model is scaled, rotated and translated in sequence from (16). These operations realize the transformation from the local coordinate system to the global coordinate system (GCS), as shown in Figure 12.

(16) $$X*TRS*P*S^{T}R^{T}T^{T}*X^{T}=0$$

where Τ is the translation matrix, i.e., $T=\left[\begin{array}{cccc} 1 & 0 & 0 & 0\\ 0 & 1 & 0 & 0\\ 0 & 0 & 1 & 0\\ T_{x} & T_{y} & T_{z} & 1 \end{array}\right]$. R is Euler rotation matrix, i.e., $R=\left[\begin{array}{cccc} c_{\beta }\cdot c_{\theta } & -c_{\beta }\cdot s_{\theta } & s_{\beta } & 0\\ s_{\alpha }\cdot s_{\beta }\cdot c_{\theta }+c_{\alpha }\cdot s_{\theta } & -s_{\alpha }\cdot s_{\beta }\cdot s_{\theta }+c_{\alpha }\cdot c_{\theta } & -s_{\alpha }\cdot c_{\beta } & 0\\ -c_{\alpha }\cdot s_{\beta }\cdot c_{\theta }+s_{\alpha }\cdot s_{\theta } & c_{\alpha }\cdot s_{\beta }\cdot s_{\theta }+s_{\alpha }\cdot c_{\theta } & c_{\alpha }\cdot c_{\beta } & 0\\ 0 & 0 & 0 & 1 \end{array}\right]$, and the symbols are defined as, $s_{\alpha }=\sin\left(\alpha \right)$, $c_{\alpha }=\sqrt{1-s_{\alpha }^{2}}$, $s_{\beta }=\sin\left(\beta \right)$, $c_{\beta }=\sqrt{1-s_{\beta }^{2}}$, $s_{\theta }=\sin\left(\theta \right)$, and $c_{\theta }=\sqrt{1-s_{\theta }^{2}}$. The angles α,β,θ correspond to the rotation angles of the *x, y* and *z* axis, respectively from the GCS to the LCS. S is a scaling matrix, i.e., $S=\left[\begin{array}{cccc} s & 0 & 0 & 0\\ 0 & s & 0 & 0\\ 0 & 0 & s & 0\\ 0 & 0 & 0 & 1 \end{array}\right]$.

**Figure 13:**
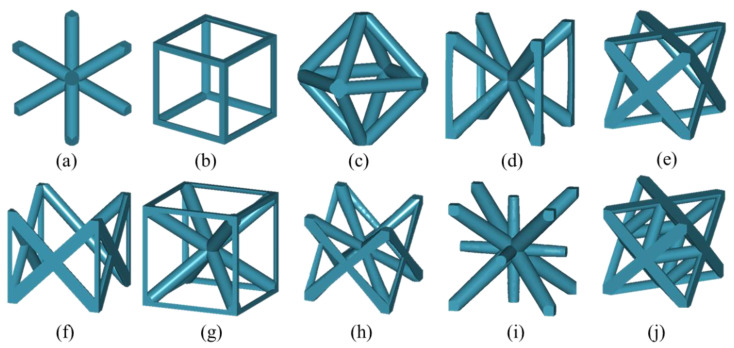
The unit cells of different shapes. (**a**) Body-centered cubic (BCC); (**b**) Simple cubic (SC); (**c**) Octahedron (OCT); (**d**) The BCC with vertical struts (BCCz); (**e**) Face-centered cubic (FCC); (**f**) The FCC with vertical struts and no struts in the horizontal plane (S-FCCz); (**g**) The union of BCC and SC (BCCzxy); (**h**) The union of S-FCC and BCC (S-FBCC); (**i**) Reinforced body-centered cubic (RBCC); (**j**) Octet-truss (OT).

Finally, several struts form a unit cell. Figure 13 shows some unit cell models with different topologies. The topological relationships at the microscopic scale are also based on the conventions in Figure 4. Designers can introduce auxiliary nodes to define more topologically complex unit cells. Moreover, it is more convenient for material evaluation at the microscopic scale due to the implicit representation of geometry and topological conventions.

## 5. Dual-Scale Shape-Material Model Representation

### 5.1. Data Structure of the DDSM

Following the framework of the dual-scale representation of model in Section 2, a new CAD data structure is proposed. For a lattice solid object, S is represented by four parts:

(17) S=f(P,M,C,U)

- Vertices array P: The array records the node information of the mesh model at the macroscopic scale. It consists of node index and node coordinates.
  (18) $$P=\left[\begin{array}{cccc} N_{1} & x_{1} & y_{1} & z_{1}\\ N_{2} & x_{2} & y_{2} & z_{2}\\ \vdots & \vdots & \vdots & \vdots \\ N_{n} & x_{n} & y_{n} & z_{n} \end{array}\right]$$
- Material library M: The material library M contains homogenized equivalent materials $M_{D}$ and unit cell fabrication materials (i.e., microscopic materials) $M_{d}$. It consists of material index and material properties. Depending on the functional requirement, the material properties on each scale can be elastic modulus E, Poisson’s ratio ν, shear modulus G and thermal expansion coefficient α, etc. Additional parameters can be attached to the columns of the array.
  (19) $$M_{D}=\left[\begin{array}{ccccc} M_{1} & E_{1} & \nu_{1} & G_{1} & \alpha_{1}\\ \vdots & & & & \\ M_{i} & E_{i} & \nu_{i} & G_{i} & \alpha_{i}\\ \vdots & & & & \\ M_{m} & E_{m} & \nu_{m} & G_{m} & \alpha_{m} \end{array}\right]$$
  (20) $$M_{d}=\left[\begin{array}{cccc} M^{\prime }{}_{1} & E_{1} & \nu_{1} & G_{1}\\ \vdots & & & \\ M^{\prime }{}_{j} & E_{j} & \nu_{j} & G_{j}\\ \vdots & & & \\ M^{\prime }{}_{t} & E_{t} & \nu_{t} & G_{t} \end{array}\right]$$
- The macroscopic model *C*: Each mesh has three attributes, the mesh identification number, eight primary nodes, and equivalent material properties. *M_i_* corresponds to equivalent material index from (19). The eight primary node storage order follows the convention in Figure 4.
  (21) $$C=\left[\begin{array}{ccccccc} C_{1} & M_{1} & N_{1} & N_{2} & \cdots & N_{7} & N_{8}\\ \vdots & & & & & & \\ C_{i} & M_{i} & N_{1} & N_{2} & \cdots & N_{7} & N_{8}\\ \vdots & & & & & & \\ C_{p} & M_{m} & N_{1} & N_{2} & \cdots & N_{7} & N_{8} \end{array}\right]$$
- The microscopic model *U*: Each unit cell is composed of *N_k_* struts and its fabrication materials. $M_{j}^{\prime }$ corresponds to fabrication material index from (20). The strut has two attributes, two nodes ID connecting the strut and strut geometric parameters. The geometry of the strut is determined by the three parameters *r*_1_, *r*_2_, *t*. All the implicit functions of struts are expressed in LCS.
  (22) $$U_{j}=\left[\begin{array}{ccccc} N_{1} & N_{2} & r_{1} & r_{2} & t\\ N_{2} & N_{2} & r_{1} & r_{2} & t\\ \vdots & & & & \\ N_{k} & N_{2} & r_{1} & r_{2} & t \end{array}\right]$$
  (23) $$U={\left[\begin{array}{ccccc} M^{\prime }{}_{1} & \cdots & M^{\prime }{}_{j} & \cdots & M^{\prime }{}_{t}\\ U_{1} & \cdots & U_{j} & \cdots & U_{m} \end{array}\right]}^{T}$$

### 5.2. Prototype Implementation

**Figure 14:**
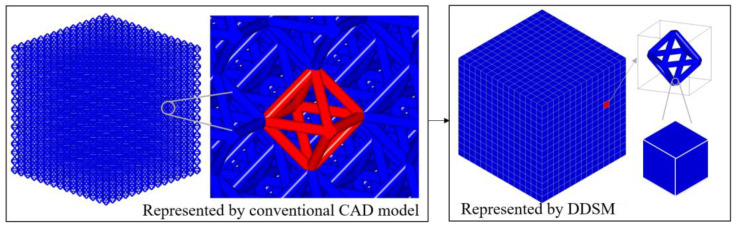
A 30 × 30 × 30 mm^3^ cubic domain consisting of octahedral unit cells.

In this section, the three benefits of the DDSM model will be discussed over the STL format. Firstly, the DDSM contains dual-material information in addition to geometric information. The STL file format represent only the geometric information of the model. Therefore, it is difficult to complete material evaluation through geometric information. In the dual-scale framework, as shown in Figure 14, the lattice structure can be viewed as volume meshes at the macroscopic scale. The CAHF data structure constructs the CAD model with complete topological relationships, which helps the equivalent material query. Moreover, the equivalent material property of unit cell takes the CAD model down from the macroscopic scale to the microscopic scale. The microscopic CAD model includes information on the geometry and manufacturing materials of the unit cell. The implicit representation can answer the question of whether the manufacturing material exists at the geometric points. 

Secondly, in terms of memory overhead, the DDSM offers complete model information with a small storage overhead. The lattice structures are usually stored in STL format. However, the STL requires to record a large amount of facet geometric information due to Delaunay triangulation. The DDSM records model geometric and material information through two scales. As shown in Figure 14, the result show that the DDSM proposed requires less than 910 KB storage space, whereas coarse STL needs 1.38 GB storage space.

**Figure 15:**
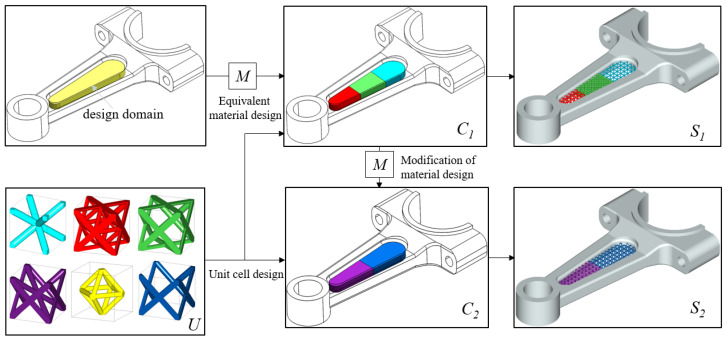
The connecting rod CAD model represented by the DDSM.

Thirdly, the DDSM reflects the user’s design intent, which makes the CAD model revisable easily. Figure 15 shows the ideal design process for the lattice structure represented by the DDSM. Specially, the middle part of the connecting rod has been designed as a lattice structure for the lightweight design. The designer can make equivalent material design to determine macroscopic CAD model, which is represented by the CAHF. The unit cell configurations are obtained from equivalent material properties in *U*. Therefore, the geometric model of the product is thus quickly customized by the DDSM as shown in *S*_1_. When the unit cell type needs to be modified in certain areas, the designers can simply modify the material distribution of target areas to obtain a new CAD model *S*_2_. Compared to the DDSM format, the STL file with only facet information is powerless in modifying model. Meanwhile, it is hard and time-consuming to model the geometric again from $S_{1}$ to *S*_2_ by the current CAD tools. 

## 6. Conclusions

This paper presents a new CAD model (DDSM) for lattice representation. The key ingredients of the CAD model are complete shape-material representation on each scale and mapping linking the two scales. Compared to the conventional CAD file format, the DDSM demonstrates advantages in terms of material information representation, memory overhead and the capture of design intent. For different functional requirements, the DDSM embodies trans-scale parallel design. Unit cell configurations at the microscopic scale determine the properties of the equivalent material. Equivalent materials reflect the geometric design intent at the macroscopic scale. 

Non-manifold geometry and implicit functions are used to construct the DDSM. The proposed CAHF verifies that it is complete and unambiguous to construct macroscopic CAD models. At the microscopic scale, the LMC is regarded as a collection of unit cells. A new implicit representation model determines the geometry and material distribution. According to the findings of this paper, the LMC represented by the DDSM meets the modern product design needs better than the conventional CAD format. Future work should include CAD model representation of unit cell incompatible connections and slicing information extraction of the DDSM for additive manufacturing.

## Figures and Tables

**Table 1:** Strut type defined by *a*.

Value of *a*	Shape
$r^{2}/\left(r^{2}-1\right)<a<k^{2}/\left(k^{2}-1\right)$	Hyperbola strut
$a=k^{2}/\left(k^{2}-1\right)$	Cone strut
$k^{2}/\left(k^{2}-1\right)<a<0$	Hyperbola strut
a=0	Parabola strut
0<a<(kr+k−r)/(kr+k−r−1)	Ellipse strut

## Data Availability

Not applicable.
